# Imaging in the diagnosis of ulnar nerve pathologies—a neoteric approach

**DOI:** 10.1186/s13244-019-0714-x

**Published:** 2019-03-20

**Authors:** Aakanksha Agarwal, Abhishek Chandra, Usha Jaipal, Narender Saini

**Affiliations:** 1 Department of Radiodiagnosis, SMS Medical College, Jaipur, Rajasthan India; 2 Department of Orthopaedics, SMS Medical College, Jaipur, Rajasthan India; 3 A 235, Shivanand Marg, Malviya Nagar, Jaipur, India

**Keywords:** Imaging in the ulnar mononeuropathy, High-resolution ultrasound of ulnar neuropathy, MR ulnar neuropathy

## Abstract

The ulnar nerve is a branch of the C8 and T1 nerve roots and arises from the medial cord of the brachial plexus. It supplies the intrinsic muscles of the hand and assists the median nerve in functioning of the flexors. Also known as the musician’s nerve, it is the second most common nerve involved in compressive neuropathy following the median nerve. Common sites of entrapment include cubital tunnel at the elbow, the ulnar groove in the humerus and the Guyon’s canal at the wrist. Patients present with altered sensation in the ulnar fourth and the fifth digit and the medial side of arm with loss of function of intrinsic muscles of the hand, the flexor carpi ulnaris and ulnar fibres of flexor digitorum superficialis in more severe cases. Diagnosis relies on clinical examination, electrodiagnostic studies and imaging findings. Plain radiographs are used to identify fracture sites, callus, or tumours as cause of compression. Technological advances in ultrasonography have allowed direct visualisation of the involved nerve with assessment of exact site, extent and type of injury. It yields unmatched information about anatomical details of the nerve. MR imaging adds to soft tissue details and helps in characterising the lesion. This pictorial review aims to illustrate a wide spectrum of causes of ulnar neuropathies as seen on ultrasound and MRI and emphasises upon the importance of imaging modalities in the diagnosis of neuropathies.

## Key points

- Over the last decade, neuromuscular ultrasound and MR neurography have emerged as useful tools for the diagnosis of peripheral nerve disorders.
- The ulnar nerve is the second most common nerve involved in compressive neuropathies of the upper limb. This pictorial review aims to demonstrate various ulnar nerve pathologies as diagnosed using imaging.
- Imaging helps in identifying the exact anatomical details about the involved nerve which provides an edge over the information provided by the electrodiagnostic tests.
- Awareness about the pathology and extent of involvement helps in proper clinical decision making and timely management.

## Introduction

Following the carpal tunnel syndrome, compressive neuropathy of the ulnar nerve at the cubital tunnel is the most common cause of compressive neuropathy [1, 2]. Conventionally, neuropathies have been diagnosed by clinical examination, Tinel’s sign and electrodiagnostic (nerve conduction velocity and electromyography) findings which provide information about the nerve involved and the possible site of injury but do not provide any accurate anatomical information. Introduction of high-frequency ultrasound probes have made direct visualisation of peripheral nerves possible, thus providing anatomical details about the nerve. Ultrasonographic findings of peripheral nerves were first reviewed by Fornage in 1988 [3]. Since then, technological advances like increased frequency and variable sizes of footprints of linear transducers have escalated the use of ultrasound in peripheral nerve pathologies. Exact site, extent and type of involvement, local cause of neuropathy, continuity of nerve and architectural distortion can be identified for accurate diagnosis and planning the management. Ultrasound is quick, cost effective, has no contraindications and provides detailed imaging of the entire length of the nerve. MR imaging adds to soft tissue details, change in muscles supplied by the affected nerve and helps in characterising the nerve lesion. In-spite of these advantages, imaging remains underutilised in cases of peripheral neuropathy. The aim of this pictorial essay is to demonstrate the use of imaging in diagnosing the ulnar nerve neuropathies due to various aetiologies. Various previous publications have mentioned methods to identify the nerve in specific anatomical locations and various peripheral nerve pathologies, centred around the Guyon’s canal and Cubital tunnel [3–7]. With this review, we provide a neoteric review which combines ultrasound and MR imaging of commonly encountered causes of ulnar neuropathies.

## Path of the ulnar nerve

**Fig. 1:**
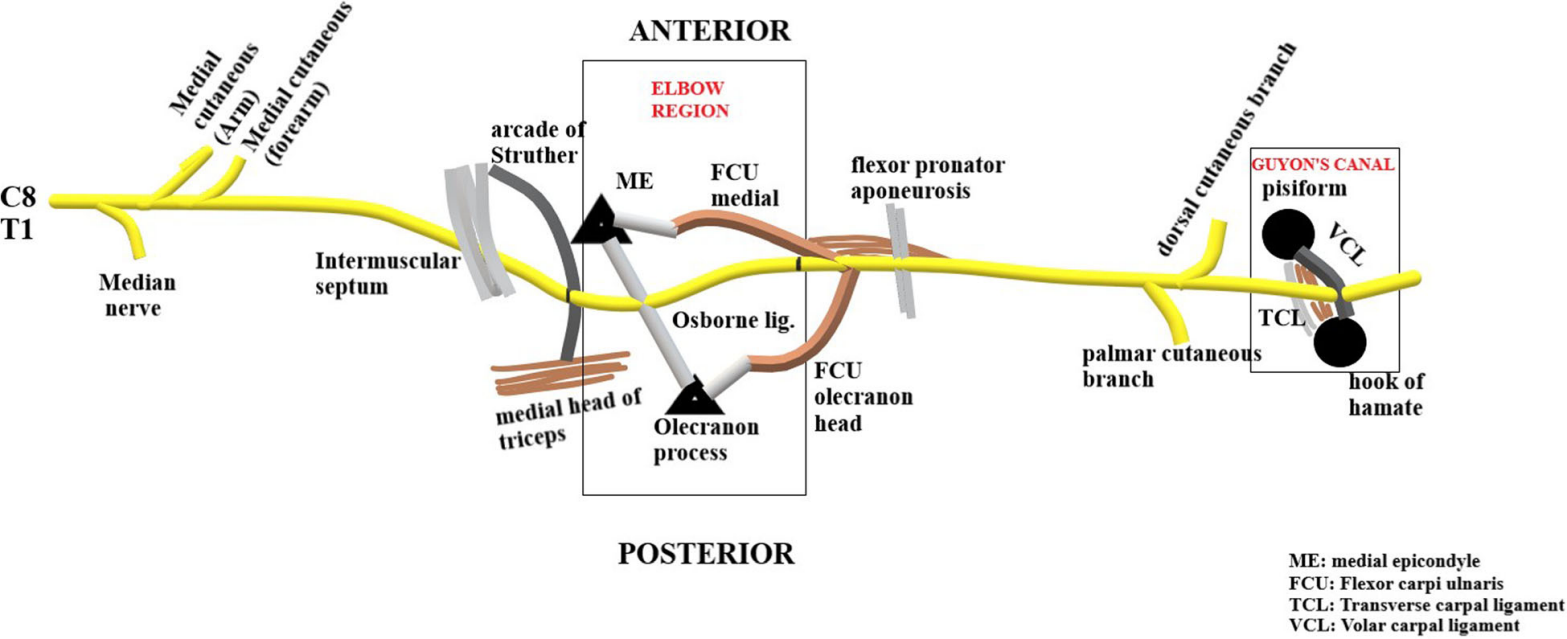
Diagrammatic representation of the pathway of the ulnar nerve from origin up to terminal bifurcation at wrist. The elbow and wrist are the most common sites of ulnar nerve entrapment

The nerve arises from the medial cord of the brachial plexus carrying fibres from the C8-T1 nerve roots. It lies posteromedial to the brachial artery in the anterior compartment of the upper arm and descends down to enter the posterior compartment by piercing the medial intermuscular septum (Fig. 1). This is a potential site of compression of the nerve under the ‘Arcade of Struther’ [8] which is described as a fibrous canal on the medial aspect of the middle- and lower-third of the arm, consisting of the medial head of the triceps brachii muscle and its aponeurotic expansion, which extends into the intermuscular septum and internal brachial ligament and covers part of the ulnar nerve. Thereafter, the ulnar nerve runs behind the medial epicondyle with the superior ulnar collateral vessels under the ‘Cubital tunnel’ [9–11]. The roof of the cubital tunnel is formed by the Osborne’s ligament or the Cubital retinaculum which is a ligament spanning from the medial epicondyle to the olecranon process, continuous with the fascia connecting the humeral and ulnar heads of the flexor carpi ulnaris (FCU). Alternatively, the roof may be formed by the anconeus epitrochearlis muscle [12]. The floor is formed by the medial collateral ligament (MCL) and elbow joint capsule, while the medial epicondyle and olecranon form the walls on either side [13]. Flexion of the elbow decreases the height, area and sagittal curvature of the tunnel, with maximum nerve compression occurring at 135° of flexion according to a study by James et al. [14]. At this level, the ulnar nerve gives off an articular branch to the elbow joint.

Thereafter, the ulnar nerve descends into the forearm, passing between the two heads of the flexor carpi ulnaris muscle (FCU) [15]. The nerve provides motor supply to the FCU and medial half of the flexor digitorum profundus. It continues distally along the ulna, lying deep to the FCU. About 5 cm distal to the medial epicondyle, the ulnar nerve pierces the flexor-pronator aponeurosis which is the fibrous common origin of the flexor and pronator muscles. Another aponeurosis extends between the flexor digitorum superficialis of the ring finger and the humeral head of the flexor carpi ulnaris, known as the ligament of Spinner, which attaches to the medial epicondyle and can cause kinking of the nerve following an anterior transposition. Near the wrist, the ulnar nerve moves lateral to the FCU, courses medial to the ulnar artery to enter the palm through the Guyon’s canal. The volar carpal ligament forms the roof of the canal while the transverse carpal ligament and the hypothenar muscles form the floor. The medial and lateral walls are formed by pisiform, pisohamate ligament, abductor digiti minimi muscle belly on the ulnar side and the hook of hamate on the radial side [16]. Near the wrist, the motor innervation of the ulnar nerve includes the thenar muscles: the adductor policis, deep head of flexor pollicis brevis (FPB); dorsal and palmar interossei and 3rd and 4th lumbricals, hypothenar muscles: abductor digiti minimi, opponens digiti minimi and flexor digiti minimi. The ulnar nerve gives dorsal and palmar cutaneous branches and a few superficial terminal branches to provide sensation to the ulnar fourth and entire fifth finger and the medial aspect of the forearm.

## Technical details of imaging

The normal peripheral nerve has a honeycomb appearance which is readily identified on imaging. The central spots are the nerve fascicles which are formed by bundles of nerve fibres surrounded by the perineurium. Each nerve fibre is also surrounded by an endoneurium which is not visible on imaging with the currently available resolution. The fascicles are in turn embedded and surrounded by epineurium which forms the sheath of the peripheral nerve [17]. Normal maximum cross-sectional area of the ulnar nerve at the level of cubital tunnel in a study by Wiesler et al. proved to be 0.065 cm^2^ [18].

### High-resolution ultrasound

**Fig. 2:**
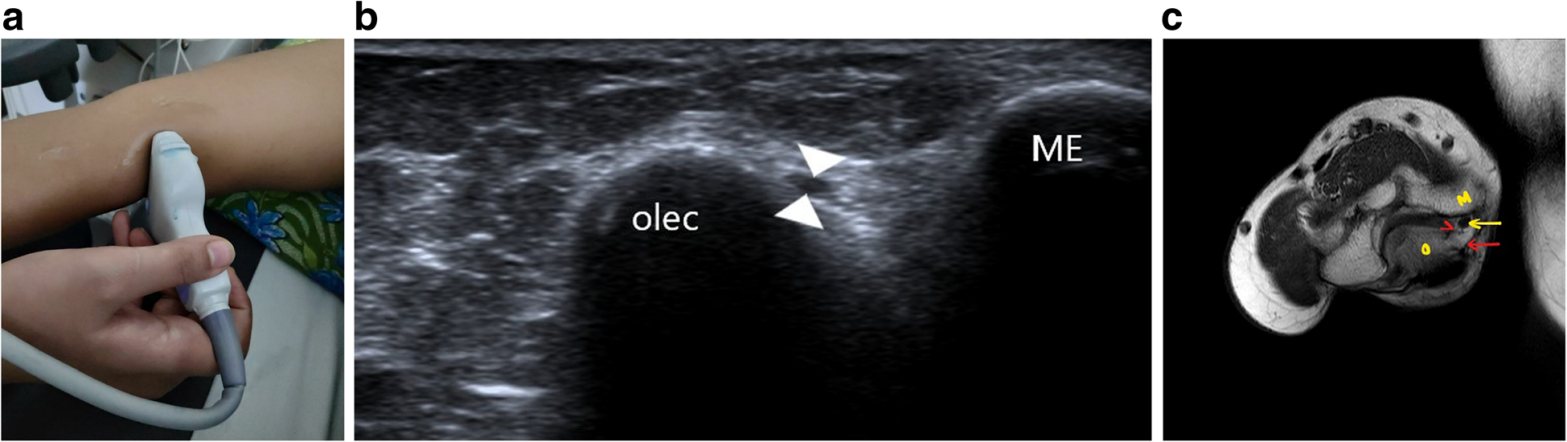
**a** Probe position for localisation of the ulnar nerve at the elbow. **b** The nerve (arrowhead) is localised in its short axis within the cubital tunnel between the medial epicondyle (ME) and olecranon process (olec). **c** MR image showing the normal ulnar nerve (yellow arrow) in the cubital tunnel: medial epicondyle (M) and olecranon process (O) forming the medial and lateral walls, Osborne’s ligament (red arrow) forming the roof and the floor being formed by the joint capsule of elbow and the medial collateral ligament (red arrowhead)

**Fig. 3:**
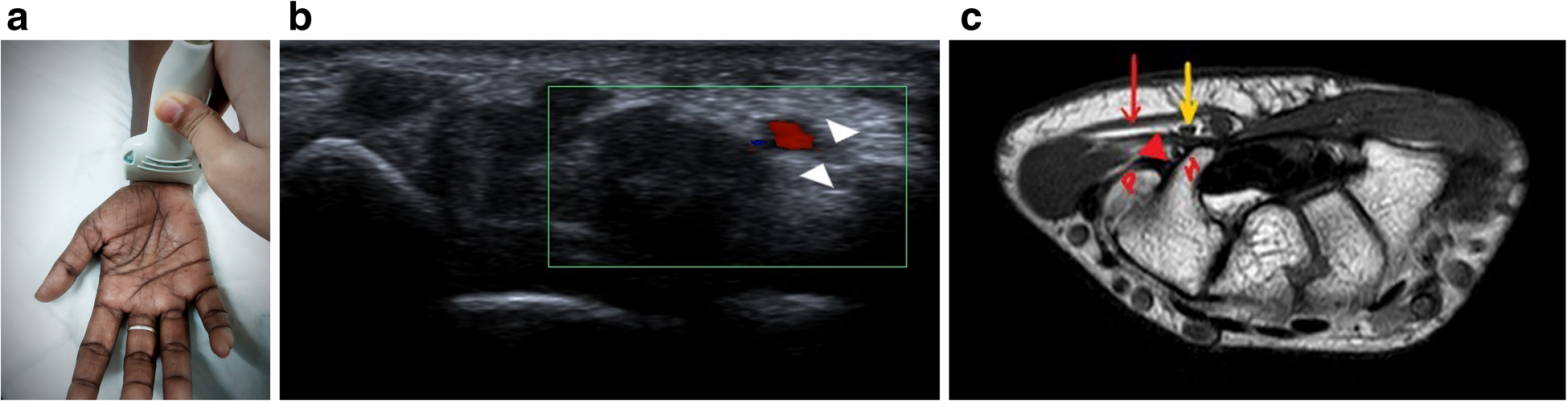
**a** Probe position for localisation of the ulnar nerve at the wrist. **b** The nerve (arrowhead) is localised in the short axis in the Guyon’s canal between the pisiform and hook of hamate, lying adjacent to the ulnar artery (colour Doppler signal). **c** MR image showing the normal ulnar nerve (yellow arrow) in the Guyon’s canal: pisiform (P) and the hook of hamate (H) form the radial and ulnar boundaries. The roof is formed by the volar carpal ligament (red arrow) while the hypothenar muscles and transverse carpal ligament (red arrowhead) form the floor

A high-frequency linear probe is required for scanning peripheral nerves. We used a probe with frequency settings to 14 Hz. The nerve should be identified in cross section as it has a characteristic honeycomb appearance which helps in demarcating it from the surrounding soft tissue. The nerve is more echogenic to surrounding muscle and less echogenic to surrounding tendon. After identifying the nerve in its short axis at predetermined anatomical locations, it can be traced proximally and distally throughout its length. The ulnar nerve is easily located at the elbow, passing under the cubital tunnel (Fig. 2) and at the wrist where it lies under the Guyon’s canal (Fig. 3). Pathological segment of the nerve will show loss of the normal honeycomb structure, decreased echogenicity, discontinuity, focal enlargement or neuroma formation. Surrounding compressive lesions, muscle and soft tissue oedema, joint effusions can also be identified.

### MR imaging

We used a 3T MRI scanner with a dedicated protocol for visualisation of the ulnar nerve. T1, T2 fat saturated, PD fat saturated sequences were acquired with slice thickness of 3 mm and interslice gap of 0.3 mm. Gadolinium-based contrast was used for post contrast images if indicated. Axial sections are of utmost importance as the characteristic honeycomb appearance is easily identified. We acquired axial sections of T1, T2 fat-saturated and PD fat-saturated sequences in addition to sagittal T1, PD fat-saturated and coronal T2 slices. Knowledge of the anatomical details of the course of the nerve is indispensable to identify the nerve in its short axis. T1-weighted images help in identification of the nerve in its short axis as the peripheral rim of fat in the nerve sheath appears hyperintense while the fascicles appear as hypointense dots within. Epineural or perineural fat remains bright on T2WI which can mask pathological changes in the nerves. Fat suppression is thus needed for identification of pathological segment of the nerve on T2 and PD sequences [19, 20]. Focal enlargement, hyperintensity and altered fascicular patterns are signs of neuropathy. The denervated muscles begin to appear hyperintense on T2-weighted images 48 h after nerve injury. These changes are seen in axonotmetic and neurotmetic injuries. MRI may not clearly differentiate between disruption injuries and contusion of nerve [21]. Muscle atrophy with persistent hyperintensity is seen in failure of regeneration [20]. Fatty infiltration is seen in chronic denervation. Orthopaedic implants may limit the use of MRI due to susceptibility artefacts.

## Clinical presentation of ulnar neuropathy

Ulnar neuropathy can result in sensory symptoms only or can cause progressively worsening motor deficit. It usually starts as altered sensation in the ulnar nerve sensory distribution, including the ulnar fourth and entire fifth finger which may progress to involve the motor fibres leading to muscular weakening and muscular atrophy over time [12, 22]. Elbow flexion can result in exaggerated symptoms as the cubital tunnel height and area decrease on flexion with resultant nerve compression [14]. Elbow flexion during sleep can result in night symptoms severe enough to cause awakening. The fibres of the intrinsic muscles of hand are located more peripherally in the nerve while the FCU and FDP fibres are more centrally placed. Chronic severe injury progressively leads to weakening of the FCU and FDP resulting in the typical ‘Ulnar claw hand’ [12]. When the ulnar nerve is divided at the wrist, only the opponens pollicis, superficial head of the flexor pollicis brevis and lateral 2 lumbricals are functioning, all of which are supplied by the median nerve.

## Aetiology of ulnar nerve pathology

Ulnar neuropathy can occur due to entrapment of the nerve at anatomical sites, chronic irritation of the nerve due to local causes or transection of the nerve following penetrating injuries.

The most common site of ulnar nerve entrapment is at the elbow, within the cubital tunnel or the epicondylar groove [23] while the second most common site is at the wrist, commonly in the Guyon’s canal [24–27].

Another common cause of ulnar neuropathy is the ‘tardy ulnar nerve palsy’ as described by Panas et al. [28–30] in which chronic nerve irritation occurs due to prior trauma or osteoarthritis.

Common sites of nerve compression include a site in the upper arm where the ulnar nerve pierces the intermuscular septum, beneath the arcade of Struther, alongside the medial epicondyle particularly in patients with cubitus valgus deformity, in the ulnar groove, within the cubital tunnel and between the two heads of FCU. More distally, it can get compressed where it exits the FCU and perforates a fascial layer between the flexor digitorum superficialis and the flexor digitorum profundus [31, 32].

Guyon’s canal is the commonest site of direct nerve trauma following penetrating injuries as the nerve is superficial in this region [27]. Three zones have been identified in relation to the ulnar nerve pathology near the Guyon’s canal—zone 1: encompassing the area proximal to the bifurcation of the ulnar nerve; zone 2: encompassing the motor branch of the nerve after it has bifurcated; and zone 3: encompassing the superficial or sensory branch of the bifurcated nerve.

Other aetiologies include compression during general anaesthesia, blunt trauma, deformities, transient occlusion of brachial artery, cigarette smoking, entrapment within scar tissue, local hematomas, subdermal contraceptive implants and following venepuncture [33–37]. Thermal and burn injuries are another cause of ulnar nerve palsy which may also include other nerves in the region of the burn. Tumour and tumour like lesions involving the ulnar nerve include peripheral nerve sheath tumours (PNSTs), fibrolipomatous hypertrophy, intraneural lipomas, intraneural ganglion, true neuromas and pseudo neuromas.

## Diagnosis of ulnar nerve pathologies

Clinical examination and electrodiagnostic studies (nerve conduction velocity (NCV) and electromyography (EMG)) are the conventional diagnostic tools in neuropathies as they provide essential information about the type of dysfunction and help in clinical monitoring. Tinel’s sign helps in assessing the possible site of injury and to assess response to treatment. Nerve injuries have been classified by Seddon in 1943 and expanded by Sunderland in 1951. Mackinnon and Dellon in 1992 added grade VI injury to Sunderland grading scheme which was defined as a mixed type of injury which denotes various types of injuries across the cross section of the nerve [38–40]. Table 1 illustrates this classification along with imaging findings in different types of nerve injuries. Imaging helps by providing unmatched anatomical details about the nerve involved. Plain radiographs show fracture sites, soft tissue swellings and orthopaedic implants. It may also demonstrate a fat stripe of lipoma or a bony lesion in proximity to the course of the nerve. High-resolution ultrasound and MRI help in direct visualisation of the nerve with anatomical delineation of the pathological segment. Hypo-echogenicity of the nerve on ultrasound and hyperintensity on MRI are markers of neuropathy. Normal nerves do not show any intra-neural vascularity. Appreciation of intra-neural vascularity is a marker of neural compression or inflammation and co-relates with severity of affection of nerve [41]. These imaging modalities also provide essential information about the surrounding soft tissue and the muscles innervated by the injured nerve.

**Table 1:**
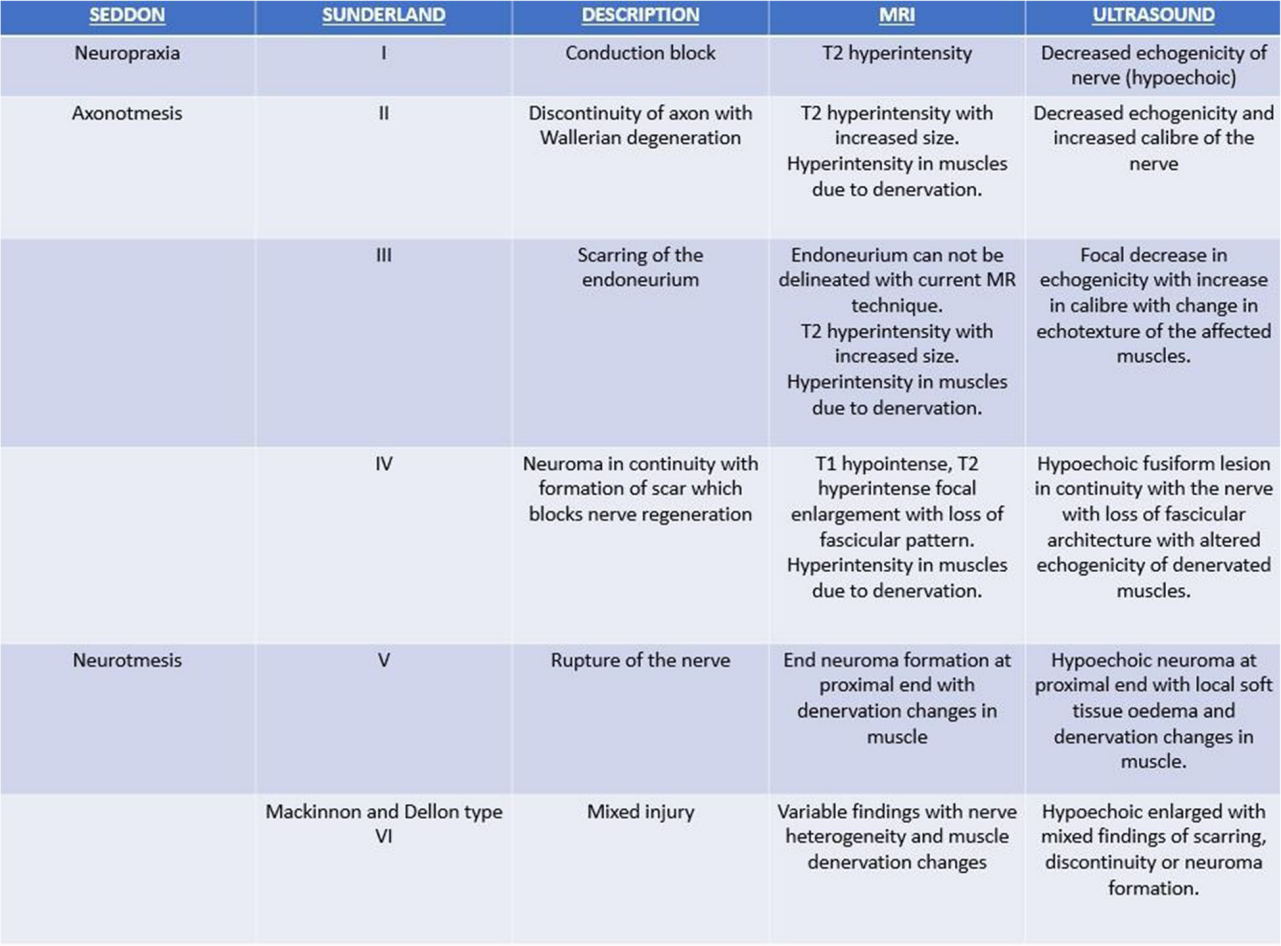
Imaging findings on high-resolution ultrasound and MR neurogram according to the nerve injury classification [49]

## Imaging findings in a wide spectrum of ulnar nerve pathologies

### Ulnar nerve compression at the elbow

#### Due to ganglion cyst

Ganglion cysts are pseudocysts with no epithelial lining of their own. These are non-neoplastic lesions filled with gelatinous material and originate from tendon sheath, ligament, bursa, joint capsule or subchondral bone. Rarely, they may present in an intramuscular location, away from the joint with no synovial communication [42, 43]. Ganglion cysts of the nerve sheath are uncommon. There are reports in the literature of ulnar nerve compression by extra-neural ganglion cysts at the elbow and less commonly in the Guyon’s canal [44].

**Fig. 4:**
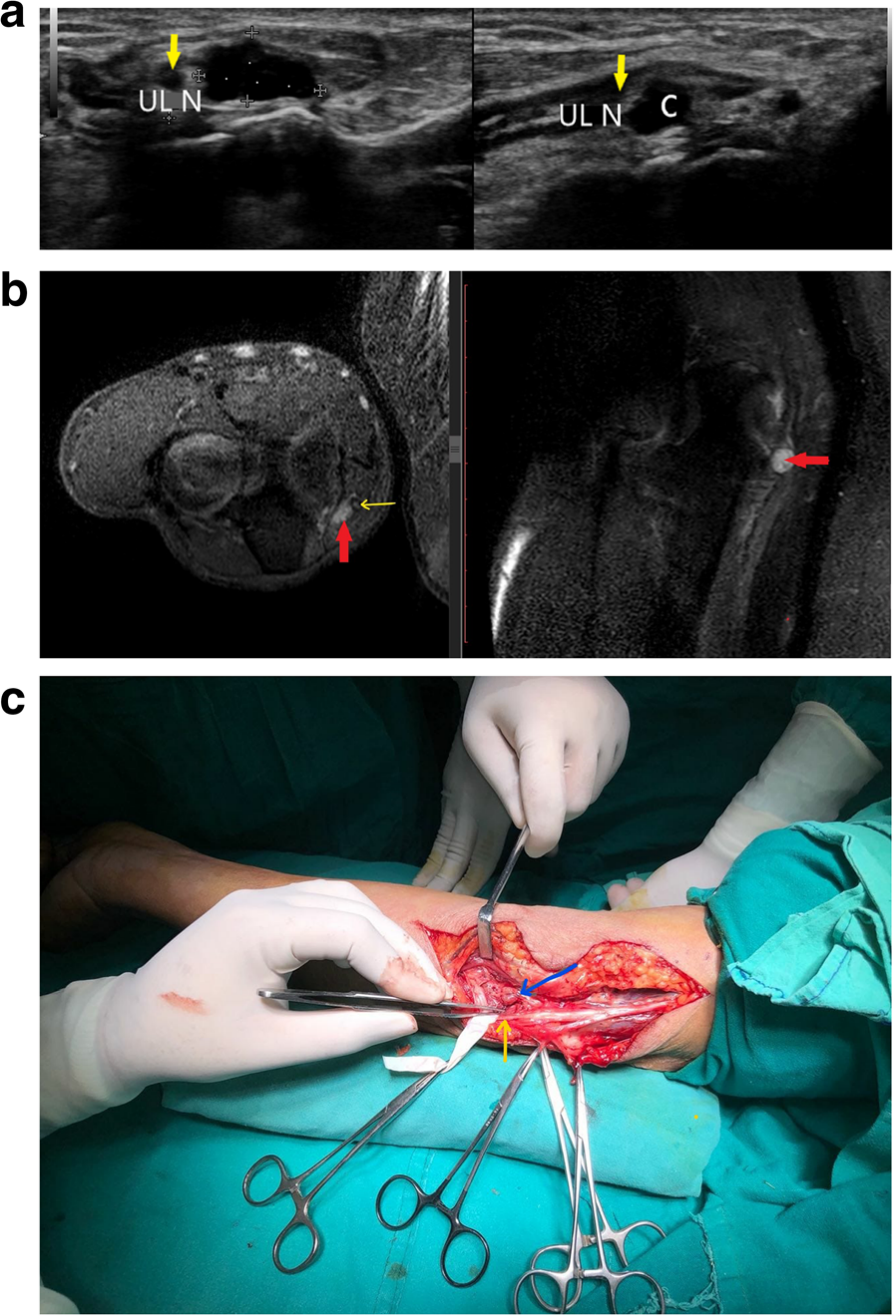
**a** Short axis and long axis view of ulnar nerve (yellow arrow) which appears hypoechoic. There is a cystic lesion (between callipers) causing direct compression over the ulnar nerve. **b** PD FS axial and sagittal image showing a hyperintense ulnar nerve (yellow arrow) getting compressed by a hyperintense cyst (red arrow). **c** Per operative picture showing the ulnar nerve (yellow arrow) and the cyst (blue arrow). Histopathology revealed it to be a ganglion cyst

Figure 4 shows a simple cystic lesion on ultrasound causing ulnar nerve compression at the elbow. The nerve was hypoechoic on HRUS and hyperintense on MRI signifying neuropathic changes. Per operatively, neural compression by a ganglion cyst lying within the cubital tunnel under the fascial sheath of the FCU muscle was confirmed.

**Fig. 5:**
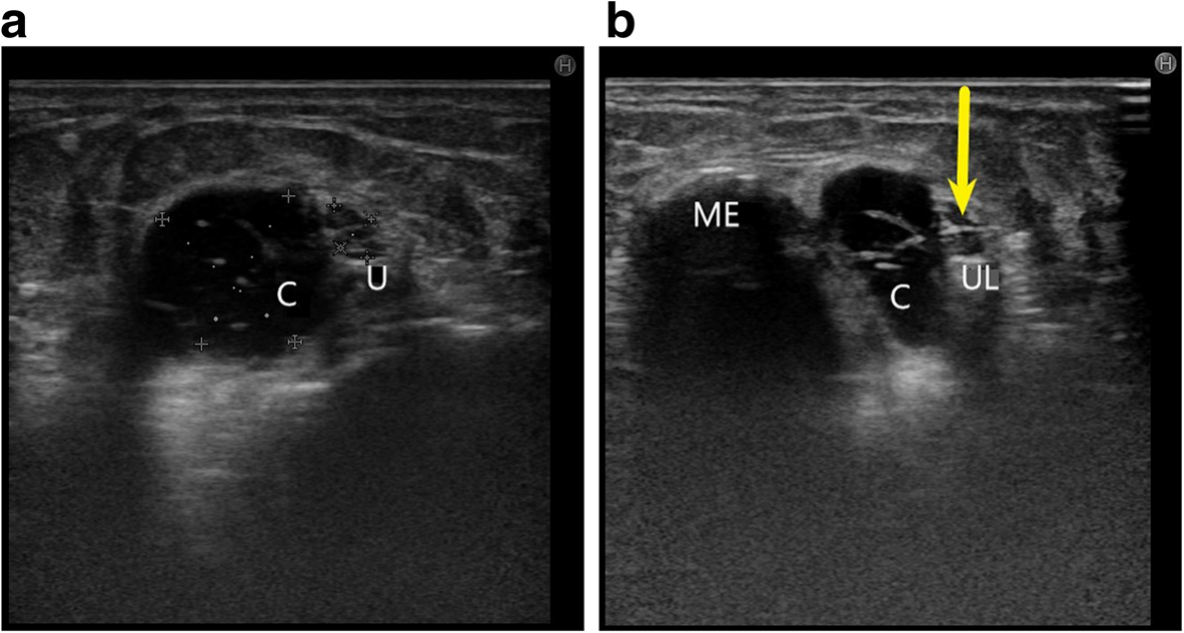
**a** Thickened and hypoechoic ulnar nerve (U between callipers) is noted getting compressed by a cystic lesion with internal echoes (C) at the level of cubital tunnel. **b** Short axis view of the ulnar nerve (yellow arrow) shows direct compression of the nerve by a cyst which was diagnosed as an infected ganglion cyst after excision

Figure 5 shows another case of ulnar nerve compression by a similar infected ganglion cyst in a patient with loss of sensations in the ulnar distribution of hand and decreased power of the intrinsic muscles of hand. The FCU supply was spared as the cyst was compressing the nerve distal to the origin of the FCU fibres.

**Fig. 6:**
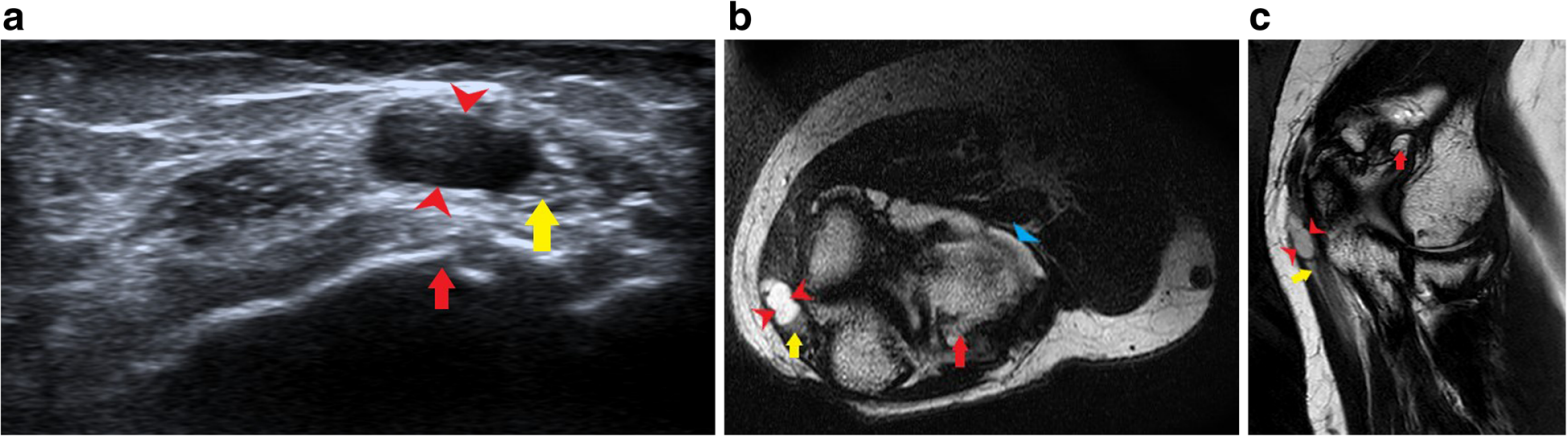
**a** Anechoic cystic lesion (red arrowheads) adjacent to the ulnar nerve (yellow arrow) at the elbow with underlying cortical erosions (red arrow). The cystic lesion appears to arise from the nerve, suggestive of an intraneural ganglion cyst. **b** T2-weighted axial section shows hyperintense cystic lesion (red arrowheads) which post operatively was diagnosed as ganglion cyst arising from the nerve sheath of the ulnar nerve (yellow arrow). The nerve is hyperintense due to compressive neuropathy. Also noted are osteophytes (red arrow) and joint effusion (blue arrowhead) suggestive of arthritic changes. **c** T2-weighted coronal image shows the cystic lesion (red arrowheads) arising from the adjacent ulnar nerve (yellow arrow) lying along the course of the nerve [54]. Also noted are osteophytes (red arrow)

Figure 6 shows a case of ganglion cyst arising from the nerve sheath which is causing compression of the nerve fibres resulting in neuropathic symptoms along the distribution of the ulnar nerve.

#### Due to compression by fracture segment

**Fig. 7:**
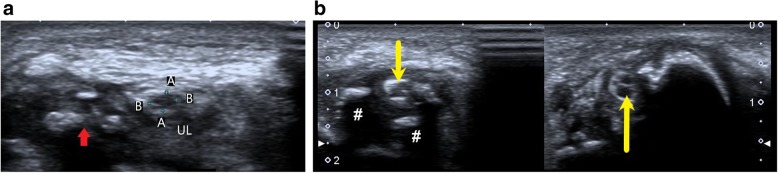
**a** Hypoechoic ulnar nerve (between callipers, marked A and B) is noted just above the elbow in a case of supracondylar fracture of humerus. The fracture fragments (red arrow) impinge upon the nerve, resulting in ulnar neuropathy. **b** Short axis view of the ulnar nerve (yellow arrow) getting compressed by the fracture fragments (#) with surrounding inflammatory exudate

Fracture of the medial epicondyle or the olecranon process of ulna can impinge upon the ulnar nerve in the cubital tunnel. This cortical breach can be well visualised on plain radiographs while compression of the nerve can be seen on HRUS. Figure 7 shows a case with ulnar compression by fracture fragment at the elbow.

#### Due to orthopaedic implants

**Fig. 8:**
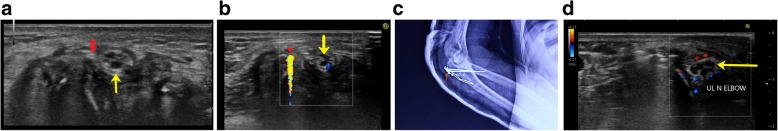
**a** Thickened hypoechoic ulnar nerve (yellow arrow) getting compressed by orthopaedic implant—K wire (red arrow). **b** Ring down artefact on colour Doppler shows the orthopaedic implant (red arrowhead) compressing the hypoechoic ulnar nerve which also shows increased vascularity suggestive of neuritis (yellow arrow). **c** Plain radiograph of the same patient shows the implant following intervention for an olecranon fracture. **d** Colour Doppler image of the same patient showing hypervascularity of the ulnar nerve (yellow arrow) which co-relates with higher grade of neuritis due to compression or inflammation [41]

Metallic K wires are frequently used in banding of fractures of the olecranon process. These may impinge upon the nerve causing chronic nerve irritation. Figure 8 shows a case with ulnar nerve impingement by a K wire used for tension band wiring of the olecranon process.

### Tardy ulnar nerve palsy

Tardy ulnar nerve palsy is a clinical condition characterised by delayed onset ulnar neuropathy due to nerve compression. It has a variety of etiological factors including callus of old healed fractures around the elbow, arthritis, effusions and synovial thickening of the elbow joint, congenital anomalies, adhesion, recurrent dislocation of the elbow, etc. Tardy nerve palsy can also result from cubitus varus deformity following old supracondylar fracture of humerus [45, 46].

**Fig. 9:**
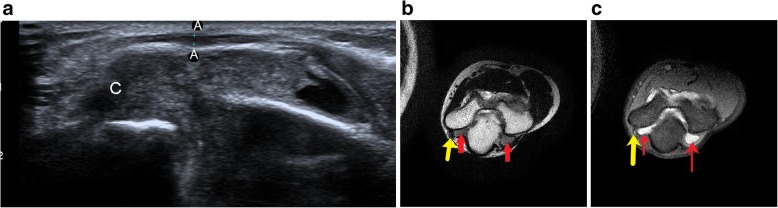
**a** The ulnar nerve (A) is of normal calibre but is displaced out of the cubital tunnel due to underlying hypoechoic collection (C) within the joint cavity. **b** T1-weighted axial image at the level of cubital tunnel shows hypoechoic ulnar nerve (yellow arrow) with surrounding isointense significantly thickened synovium (red arrows). **c** PD FS image shows isointense ulnar nerve (yellow arrow) with normal calibre with surrounding hyperintense synovial thickening (red arrows) and minimum joint effusion. On further laboratory investigation, the patient was diagnosed to be a case of rheumatoid arthritis

Figure 9 shows a case with ulnar neuropathy due to underlying joint effusion and synovial thickening in a patient with juvenile rheumatoid arthritis. The ulnar nerve was displaced out of the cubital tunnel due to underlying soft tissue.

**Fig. 10:**
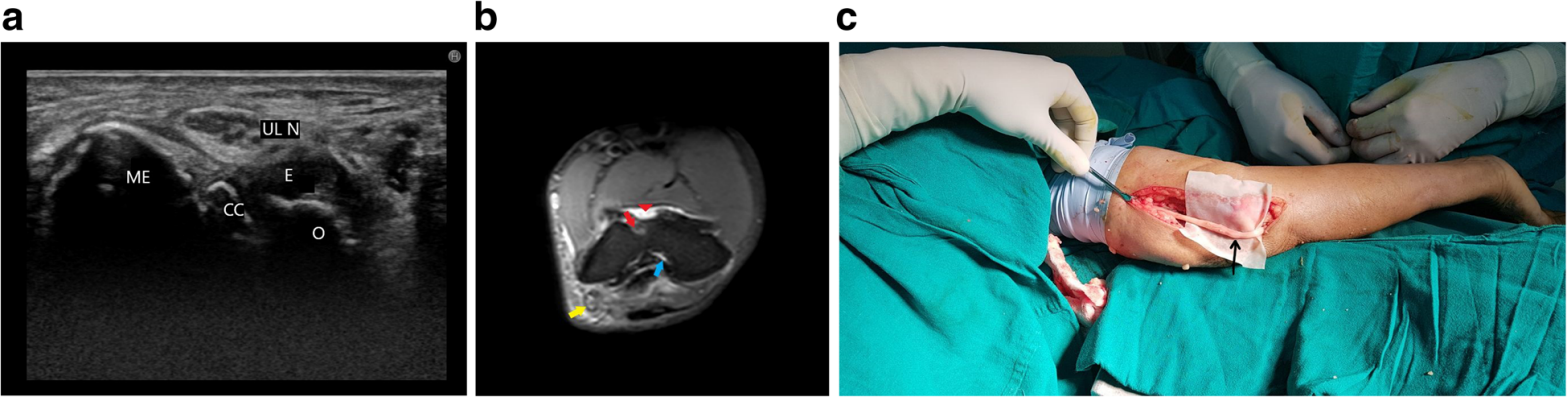
**a** Grey scale ultrasound image shows a hypoechoic, significantly thickened ulnar nerve (ULN) in the cubital tunnel with underlying joint effusion (E) and cortical irregularity (CC). The medial epicondyle (ME) and olecranon process (O) forming boundaries of the cubital tunnel are also seen. **b** T2-weighted MR image of the patient shows hyperintensity of the ulnar nerve (yellow arrow) with surrounding arthritic changes—subchondral cyst (red arrow), marrow oedema with cortical irregularity (blue arrow) and joint effusion (red arrowheads). **c** Intra operative picture of the patient showing the thickened ulnar nerve (black arrow)

Age-related arthritic changes, as seen in Fig. 10 are another probable cause of ulnar neuropathy in patients with tardy ulnar nerve palsy. Also seen is increased neural vascularity.

**Fig. 11:**
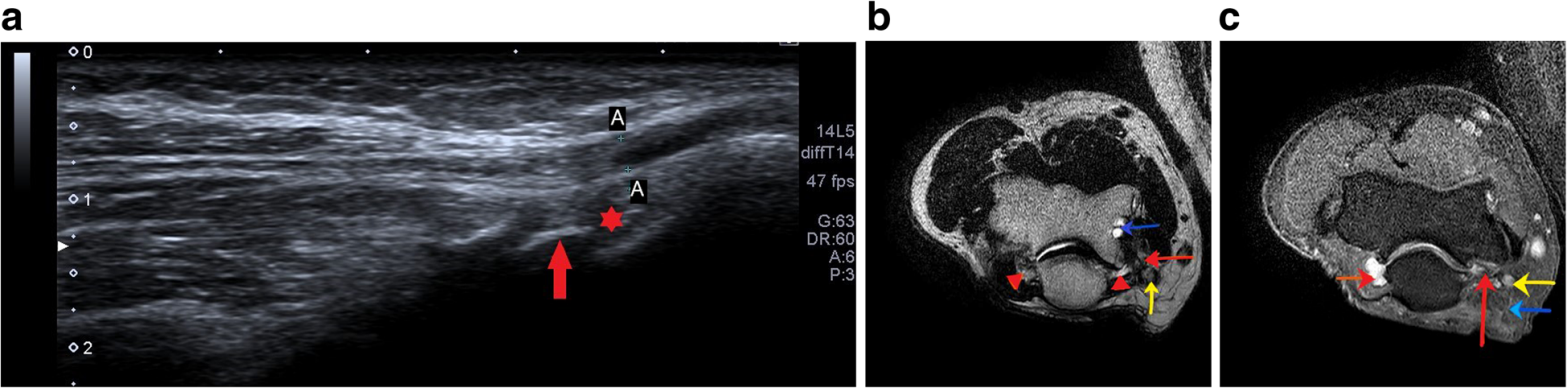
**a** Hypoechoic ulnar nerve (A) at the elbow with underlying synovial thickening (red star) and cortical irregularity (red arrow). **b** Thickened hyperintense ulnar nerve (yellow arrow) with surrounding synovial thickening (red arrowhead) and joint effusion on T2-weighted MR image. Also noted is subchondral cysts (blue arrow) and cortical irregularity (red arrow). **c** PD FS image shows hyperintense ulnar nerve (yellow arrow), joint effusion (red arrow) and panniculitis (blue arrow), all features of chronic inflammation

Age-related joint effusion, subchondral cysts, and synovial thickening can cause displacement of the ulnar nerve out of the cubital tunnel, as seen in Fig. 11.

**Fig. 12:**
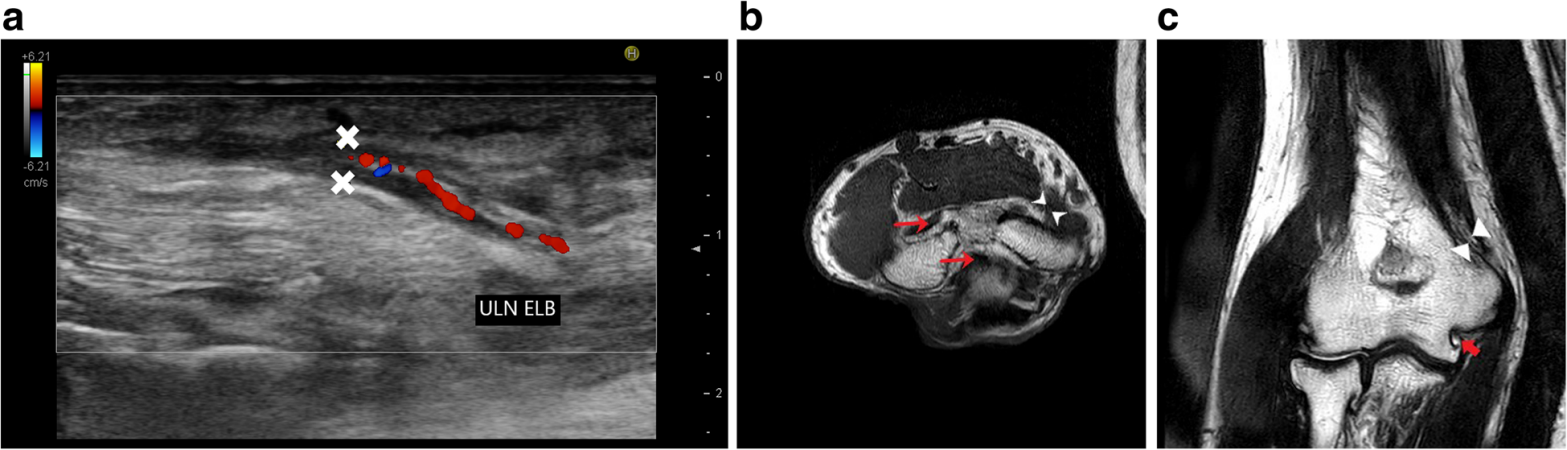
**a** Thickened, hypoechoic ulnar nerve with increased intra neural vascularity on colour Doppler, signifying ulnar neuritis. **b** Ulnar nerve (white arrow head) is hypointense and located anteromedial to the normal location anterior to the medial epicondyle (ME) in a case with anterior intramuscular transposition of ulnar nerve. There is also associated joint effusion (red arrow) which appears hypointense on this T1-weighted image. **c** Ulnar nerve (white arrow head) appears hyperintense and thickened on T2 coronal image lying above the medial epicondyle, passing lateral to it instead of its normal location in the cubital tunnel. Also noted are osteophytes (red arrow)

Figure 12 shows another case with history of tardy ulnar nerve palsy. The nerve was transposed anteriorly to relieve compression. The patient developed neuritis post operatively which was subsequently managed with steroids and analgesics.

## Ulnar neuropathy in leprosy

One of the principal causes of ulnar neuropathy in the Indian subcontinent is infection by lepra bacilli. Nerve involvement in leprosy may vary from involvement of an intradermal nerve in the cutaneous patch to a major lesion in the peripheral or the cranial nerve trunk [47]. Nerve damage in leprosy may present itself as silent neuropathy without overt signs and symptoms or as clinically manifest disease which may present as weakness, atrophy or contracture.

**Fig. 13:**
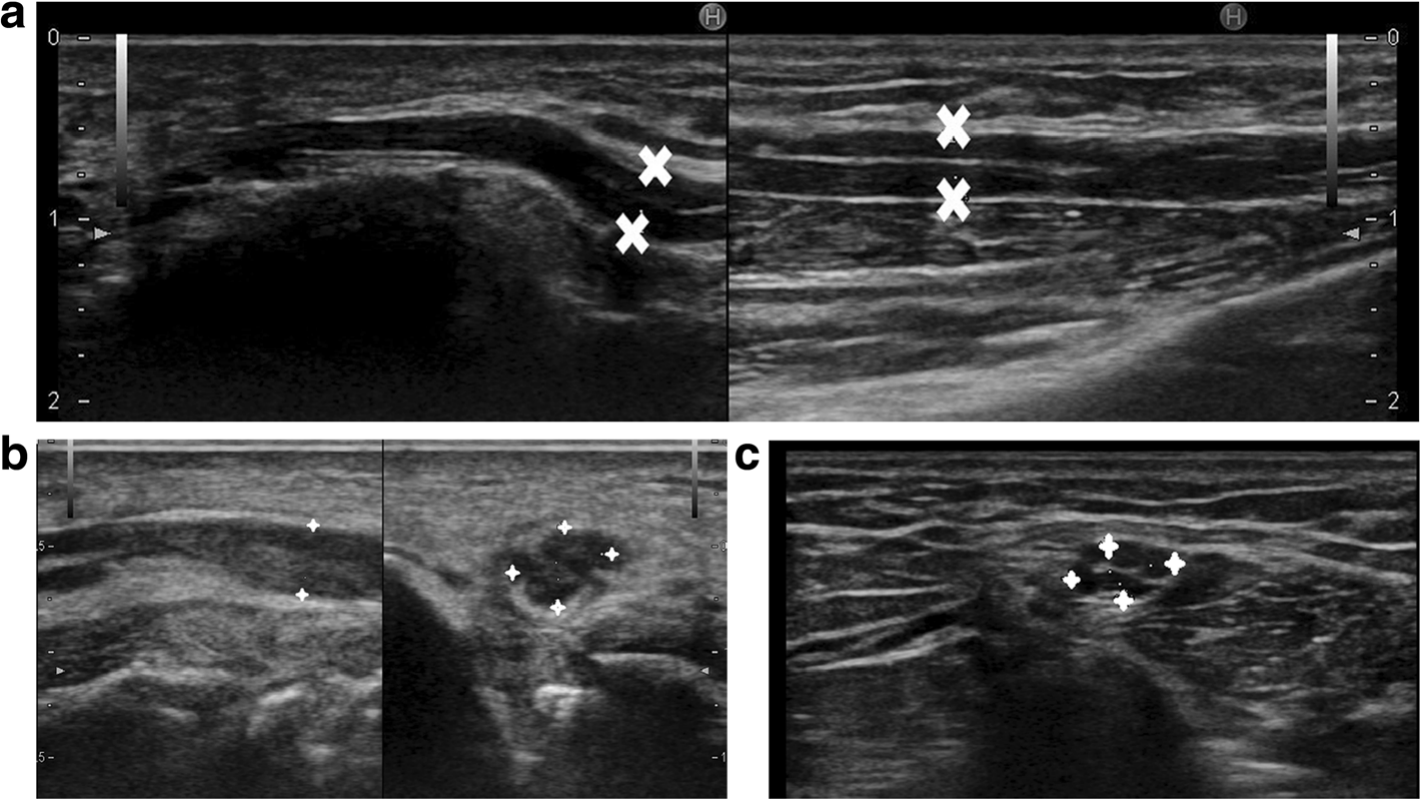
**a** A patient with lepromatous leprosy and paraesthesia in ulnar distribution with a hypoechoic ulnar nerve (between callipers). **b** Another patient with more advanced nerve damage, as evident by loss of fascicular architecture and irregularly, thickened ulnar nerve (between callipers). **c** Hypoechoic, thickened ulnar nerve (between callipers) with swollen appearance of intraneural fascicles

Imaging features may range from a hypoechoic nerve with loss of fascicular architecture to frank abscess formation and intra neural hypervascularity. Figure 13 shows cases of ulnar nerve involvement in different stages of leprosy.

## Traumatic ulnar nerve palsy at wrist

**Fig. 14:**
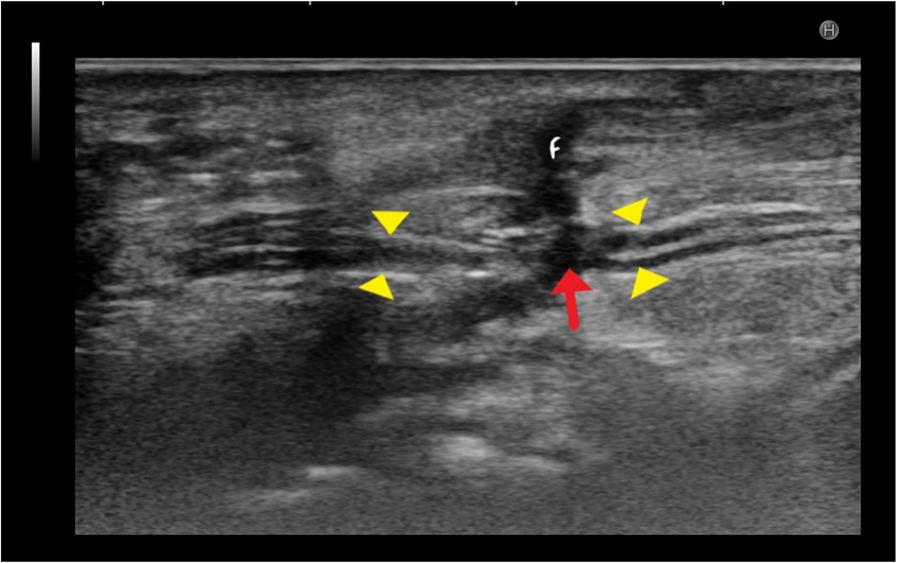
Hypoechoic ulnar nerve (between yellow arrowheads) is noted at the wrist in a patient with penetrating injury to the forearm. The nerve is discontinuous (red arrow) with fluid filled (F) tract extending from skin to the nerve

**Fig. 15:**
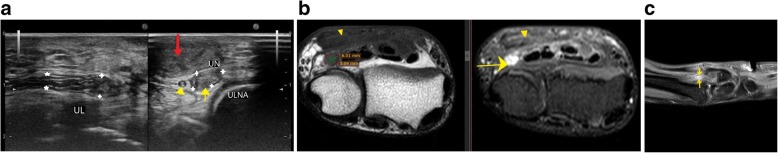
**a** Hypoechoic ulnar nerve (between white stars and crossheads) is noted in a case with glass cut injury to the wrist. At the site of injury, there is formation of neuroma in continuity (yellow arrow) characterised by thickened, hypoechoic focal area with loss of fascicular architecture. Also noted is soft tissue swelling (red arrow). Yellow arrowhead marks the ulnar artery. **b** T1- and T2-weighted images show the ulnar nerve neuroma which is hypointense on T1 and hyperintense on T2 (between callipers and yellow arrow respectively) with associated soft tissue oedema. **c** Sagittal T2-weighted image shows hyperintense neuroma in continuity (yellow arrows) along the ulnar nerve

The ulnae nerve has a superficial course near the wrist where it passes under the Guyon’s canal. It can be commonly injured in penetrating injuries to the wrist. Figure 14 shows a case with discontinuity in ulnar nerve following a penetrating injury to forearm. Another case with glass cut injury shows a neuroma in continuity in the ulnar nerve in Fig. 15 along with soft tissue oedema.

## Peripheral nerve sheath tumours involving the ulnar nerve [48, 49]

**Fig. 16:**
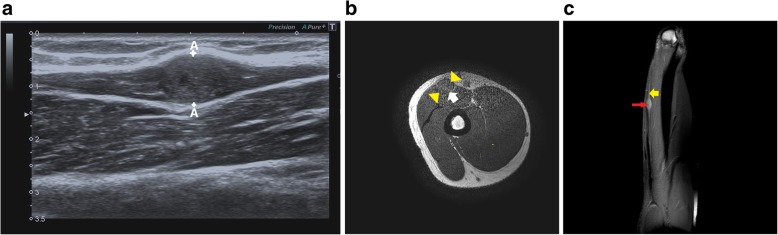
**a** Grey scale image showing characteristic fusiform swelling in continuity of the ulnar nerve, suggestive of peripheral nerve sheath tumour. **b** Hypointense lesion in continuity with ulnar nerve with fascicular pattern (yellow arrowheads) is seen with surrounding thin rim of fat (white arrow)—the split fat sign. **c** PD FS image shows a fusiform swelling which is hyperintense (red arrow) with internal hypointense areas, suggestive of collagenous stroma. Also noted is the ulnar nerve (yellow arrow) forming a tail. **d** Post contrast T1 FS image shows vivid enhancement in the PNST, differentiating it from a neuroma

**Fig. 17:**
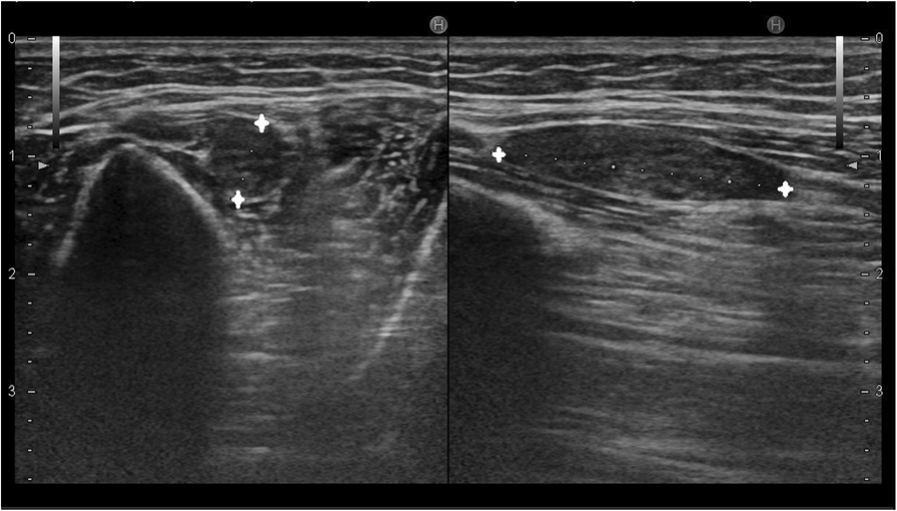
Grey scale image showing characteristic fusiform swelling in continuity of the ulnar nerve (between callipers). Many similar lesions were noted along the radial and the median nerve, bilaterally. The patient was a known case of neurofibromatosis

Peripheral nerve sheath tumours (PNSTs) include neurofibroma and schwannoma which may be benign or malignant. Deep-seated neurofibromas may have neurological symptoms while superficial neurofibromas are usually small painless masses. Plain radiography is usually normal in PNST while high-resolution ultrasound shows a hypoechoic fusiform mass in continuity with the parent nerve. MRI is the imaging modality used to differentiate between PNST and traumatic neuromas. PNST are characterised by T1 iso-hypointense, T2 hyperintense fusiform mass in continuity with the nerve which may show low signal intensity in the centre on T2-weighted images corresponding to the higher fibro-collagen content in the centre which co-relates with the organised histological architecture. Another sign is the split fat sign best seen on T1-weighted images wherein the hyperintense fat around the fusiform mass suggests intermuscular location of the lesion. Fascicular pattern may be detected within the mass which also supports its neurogenic origin [48]. It has been suggested that post contrast scans show enhancement in PNST while there is no enhancement in traumatic neuromas which form due to regeneration and scarring of the nerve [48]. However, a study by Ahlawat et al. suggests that absence of target sign and history of trauma are the most reliable signs of a traumatic neuroma. Their study showed enhancement in 88% of traumatic neuromas, which might be attributed to a broken blood-nerve barrier [50]. Figure 16 shows a PNST in continuity with the ulnar nerve in the arm in a patient with soft tissue swelling and no neural symptoms. Neurofibroma in ulnar nerve is seen in Fig. 17 in a patient with neurofibromatosis.

## Management

Conservative treatment of ulnar neuropathy focuses on pain relief, inflammation reduction, and rehabilitation. Patients are advised to avoid aggravating activities, use night splints and use NSAIDs and steroids to decrease inflammation [12, 15]. Surgery is indicated when conservative management fails or for decompression and in cases of primary repair of the nerve.

## Further advances in MRI

Diffusion tensor imaging and tractography are now being used for peripheral nerve pathologies. Jengojen et al. [51] used diffusion tensor imaging and tractography to assess acute changes in radial and median nerve following compression and demonstrated changes in radial nerve metrics. Razek et al. [52] carried out a prospective study in 39 patients with mild to moderate carpal tunnel syndrome and found positive correlation between findings of diffusion tensor imaging, electrodiagnostic tests and clinical assessment.

Neuropathic segments tend to have a lower fractional anisotropy value as compared to the normal segment [53].

## Advantages and limitations of ultrasound

Use of high-resolution ultrasound in imaging of peripheral neuropathy is a relatively new use of this age-old modality. Ultrasound is a rapid, cost effective, widely available modality with no contraindications. It allows quick assessment of the nerve throughout its length and provides unmatched data about anatomical details of the affected nerve. Technical issues like inability to image through bone, orthopaedic implants, poor resolution of deeper nerves with higher frequency transducers, suboptimal scan in patients with higher fatty tissue due to increase in depth and similar echogenicity limit its use to a certain extent. Operator dependence and need for in-depth knowledge of the anatomical details is another limiting factor.

## Conclusion

Ulnar nerve is the second most common nerve involved in compressive neuropathies of the upper limb. Patients presenting with symptoms of ulnar nerve palsy have been conventionally diagnosed using clinical and electrodiagnostic findings. Use of high-resolution ultrasound and MRI will usher in a new era of multimodality approach in the diagnosis and treatment of nerve pathologies. It allows direct visualisation of the nerve with precise anatomical details and points towards the aetio-pathogenesis of the palsy, facilitating better clinical decision making and patient management. This pictorial essay describes the hallmark of imaging findings in a wide spectrum of ulnar nerve pathologies as diagnosed using imaging modalities.
